# A Social Network Analysis of the *Western Journal of Emergency Medicine* Special Issue in Educational Research and Practice

**DOI:** 10.5811/westjem.2020.7.46958

**Published:** 2020-10-08

**Authors:** John W. Cyrus, Sally A. Santen, Chris Merritt, Brendan W. Munzer, William J. Peterson, Jeff Shockley, Jeffrey N. Love

**Affiliations:** * Tompkins-McCaw Library for the Health Sciences, Virginia Commonwealth University, Research and Education Department, Richmond, Virginia; † Virginia Commonwealth University School of Medicine, Department of Emergency Medicine, Richmond, Virginia; ‡ Alpert Medical School of Brown University, Departments of Emergency Medicine & Pediatrics, Providence, Rhode Island; § University of Michigan, Department of Emergency Medicine, Ann Arbor, Michigan; ¶ Virginia Commonwealth University, School of Business, Department of Supply Chain Management and Analytics, Richmond, Virginia; || George Washington University, Department of Emergency Medicine, Washington, DC, Maryland

## Abstract

**Introduction:**

Scholarship and academic networking are essential for promotion and productivity. To develop education scholarship, the Council of Emergency Medicine Directors (CORD) and Clerkship Directors of Emergency Medicine (CDEM) created an annual Special Issue in Educational Research and Practice of the Western Journal of Emergency Medicine. The objective of this study was to evaluate the network created by the special Issue, and explore changes within the network over time.

**Methods:**

Researchers used bibliometric data from Web of Science to create a social network analysis of institutions publishing in the first four years of the special issue using UCINET software. We analyzed whole-network and node-level metrics to describe variations and changes within the network.

**Results:**

One hundred and three (56%) Accreditation Council for Graduate Medical Education-accredited emergency medicine programs were involved in 136 articles. The majority of institutions published in one or two issues. Nearly 25% published in three or four issues. The network analysis demonstrated that the mean number of connections per institution increased over the four years (mean of 5.34; standard deviation [SD] 1.27). Mean degree centralization was low at 0.28 (SD 0.05). Network density was low (mean of 0.09; SD 0.01) with little change across four issues. Five institutions scored consistently high in betweenness centrality, demonstrating a role as connectors between institutions within the network and the potential to connect new members to the network.

**Conclusion:**

Network-wide metrics describe a consistently low-density network with decreasing degree centralization over four years. A small number of institutions within the network were persistently key players in the network. These data indicate that, aside from core institutions that publish together, the network is not widely connected. There is evidence that new institutions are coming into the network, but they are not necessarily connected to the core publishing groups. There may be opportunities to intentionally increase connections across the network and create new connections between traditionally high-performing institutions and newer members of the network. Through informal discussions with authors from high-performing institutions, there are specific behaviors that departments may use to promote education scholarship and forge these new connections.

## INTRODUCTION

For educators, publication is important for both the dissemination of educational innovation and academic promotion. Research collaboration between institutions improves circulation and generalizability, reflecting a growing trend for joint research among academic scholars and institutions.^1^ For any research community the knowledge-creation process depends on researchers’ collective ability to combine and integrate the findings from previous studies to advance new incremental knowledge in that area. Education research and scholarship are essential for the dissemination of innovative educational practices. In the recent past there has been an emphasis among academic institutions to focus on educational requirements of certifying organizations and financial outcomes with less emphasis on such things as scholarly teaching and research.^2^–^4^ The *Western Journal of Emergency Medicine* (WestJEM), Council of Emergency Medicine Directors (CORD), and the Clerkship Directors of Emergency Medicine (CDEM) came together in 2015 to create a Special Issue in Educational Research and Practice. This special issue provides the opportunity for EM researchers to collaborate and disseminate educational innovations.

In this study we sought to understand the network of authors’ institutions publishing in the special issue through social network analysis (SNA), a strategy used to investigate the social structures of groups or individuals.^5^ SNA conceptualizes a network using the ties (edges) that connect its members (nodes) by focusing on attributes of the relationship.^6^ SNA has been used in medical education to analyze research topics and trends, the dissemination of educational innovations, communities of practice, and scholarship networks.^7^,^8^ This tool captures quantitative aspects of the patterns of relationships, which allows for comparisons between different groups and network structures. When compared over time, SNA can show changes in relationships between members of a network.

Co-authorship networks are a type of social network that may help to explain the latent structure of particular scientific inquiry or the status of individual authors of research. These networks also have the potential to identify high productivity institutions, aiding in the discovery and dissemination of best practices strategies for promoting educational scholarship. The objective of this study was to evaluate and map this network of education scholars publishing in the special issue and measure characteristics of the network to assist faculty in establishing robust publishing connections.

## METHODS

### Data Collection

To assess social connectivity among authors and institutions published in the first four CORD/CDEM special issues we collected and analyzed bibliometric data as described previously.^9^ Publication data were exported from Web of Science, and the authors’ institutional affiliations were collapsed so that multiple names for one institution were grouped into a single identifier. We used institutional identifiers to calculate the number of articles with authors from more than one institution. The following data were abstracted for all articles appearing in the 2015, 2017, 2018 and 2019 WestJEM special issues: author(s); article title; year of publication; digital object identifier; and the times cited within the Web of Science (Clarivate Analytics, clarivate.com); authors’ affiliations; article type (original research, commentary, education innovation, etc); number of institutions represented by authors; and whether or not data were gathered from one or multiple institutions.

**Population Health Research Capsule**

What do we already know about this issue?

*The ability of the WestJEM Special Issue in Educational Research and Practice to encourage and connect scholars across institutions is not yet known*.

What was the research question?

What are the characteristics of the social network of institutions created by the special issue?

What was the major finding of the study?

*An increasingly diverse group of institutions is represented in the network with a core of schools publishing in consistent groups*.

How does this improve population health?

*There is opportunity to increase education research collaboration by intentionally expanding the network to include new institutions and encouraging new groupings of institutions on publications*.

### Data Analysis

We used the institution and co-authorship data to analyze the social network associated with each year of the Special Issue in Educational Research and Practice as well as all four years combined. The software UCINET (Analytic Technologies, analytictech.com) was used to conduct a SNA of the *WestJEM* Special Issues. UCINET allows the analysis of a social network through whole-network and node-level metrics as well as visual representation of the network. Whole-network and node-level metrics are used to describe variations in the network in each of the four years and across all years while the sociogram depicts the extent of the network created by all of the special issues. Institutional review board approval was not required as this is based on publicly available data and not considered to be human subjects research. Specific metrics of interest at the network and node (institution) levels are included in Table 1.

## RESULTS

**Figure 1:**
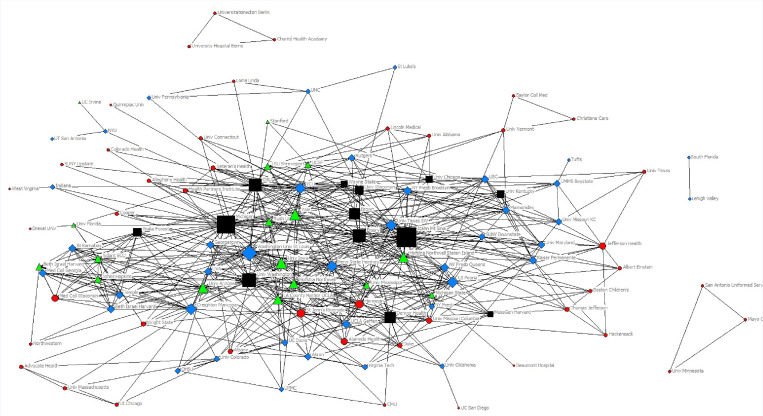
Sociogram of networked institutions from the first four years of the *West*JEM Special Issue in Educational Research and Practice. Circles represent institutions that published in a single issue. Diamonds represent institutions that published in two issues. Triangles represent institutions that published in three issues. Squares represent institutions that published in all four issues. The node size is weighted by the number of connections (degree) per node (reported for select institutions in Table 3 in the “All Years” column).

Over four years of the *West*JEM CORD/CDEM special issues, authors from 122 institutions contributed to 136 articles that were included in this analysis; a description of this dataset is published elsewhere.^9^ Of the 122 institutions that published in a special issue, 41.8% (51) published in a single year, 33.6% (41) published in two years (consecutive or not), 13.9% (17) published in three years, and 10.6% (13) published in all four years. Fifty-six percent (76) of the publications in the special issues included authors from more than one institution with a low of 42% (14) in 2015 and a high of 69% (25) in 2017. In analyzing the network created by the special issues, Figure 1 represents the relationship between institutions across all four years.

### Network-wide metrics

#### Density

Network density is a ratio measure that compares the number of actual connections between institutions in the network to the total possible *potential* institutional connections that make up the network of scholarship. The resulting score can range from 0–1. In each of the four years analyzed, and in the cumulative analysis, network density remained fairly constant across the special issues, ranging from 0.08–0.1 (mean score of 0.09). (For whole-network metrics, see Table 2.) Given that the network density score remained about .08 across all publications and years, this would imply that there was no observed expansion in collaboration between the different institutions making up the scholarship network.

#### Degree Centralization

Degree centralization measures to what extent there are a small number of highly centralized nodes (institutions) that make up the global network of special issue publications (answering the question: how centralized is the network?). The score is a ratio that compares the actual sum differences between the individual institution’s degree centrality score and the maximum degree centrality score in the network. As such, the resulting measure can range from 0–1 in the global network, where a score closer to 0 would represent a global network where all institutions are on more equal footing, whereas a larger score would indicate a network where fewer institutions were more central to the network. Overall, it appears that degree centralization was low in each of the years of the special issues (average 0.28 across four issues). However, as noted in Table 2, in the 2018 and 2019 issues, there was greater participation by a more diverse set of institutions than was seen in the earlier issues.

### Node-level Metrics

#### Degree Centrality

Degree centrality for a particular institution represents the importance of a particular institution in the network (ie, which institutions are in the center). For each institution, we calculated the degree centrality score for that institution, which is simply the sum total of the number of connections that a particular institution has to other institutions making up the network of scholarship. Three institutions placed in the top three in terms of degree centrality within the network most years (Michigan, Mt. Sinai, and Ohio State). There was considerable variation within degree centrality each year for each institution (see Table 3). With the exception of 2018, a year in which Yale did not publish in the special issue, the average degree of the network nodes increased between the initial issue and the most recent (eg, 3.48 in 2015 to 6.17 in 2019, mean 5.34).

#### Betweenness Centrality

Betweenness is another measure of centrality importance based on where a particular institution stands as a crossover point for shortest paths between all the other nodes in the entire network. The betweenness centrality score for an institution, therefore, is the number of the shortest paths that pass through that institution in the network of scholarship. The top five institutions based on betweenness scores for all four years combined were Michigan, Mt. Sinai, Ohio State, University of Washington, and Yale (see Table 3). These institutions also had authors publish in either three or four years of the special issues. While the node with the highest betweenness score varied from year to year, the same group of five institutions remained important actors in the network across the four years.

## DISCUSSION

Social network analysis serves as a useful method for investigating characteristics of the *West*JEM Special Issue in Education and Research and Practice network as it highlights key players within the network and trends within each year and across multiple years. SNA allows observation and mapping of the characteristics, connections, and frequency of interactions in the author network. This study found that the special issues represent a diverse network of authors and institutions. The network was diverse in the individual institutions represented in the issues and new institutions being introduced to the network as well as some variability of the authorship groups. In other words, often papers included different authors from different institutions and a different group of authors for other papers. Still, there were a small number of institutions that published in consistent author groups, without introducing new members to that group, and were more highly connected to the rest of the network.

Social network analysis focuses on the interactions between the members of the network.^12^ The analysis provides information about how members interact with one another and what is the level of connectedness.^13^ In the network, every network member, is not tied to every other node. There may be clusters of densely knit connections, while other members may only be connected from the periphery through a central member. The relationships reflect a flow of interactions and opportunities. It is these varying degrees of closeness, or connectedness, that determine the influence that node may have on others. Social network analysis has been widely applied across other fields and in a few studies on medical networks to describe the relationships of the members.^7^,^8^,^14^,^15^

As indicated in Table 2 by the network density remained low and did not change significantly over four years. One would expect that if the same people are in a network and developing new relationships over time, then density would increase as more connections are made. Rather, there was no change in density reflected here, which suggests that relationships are stable and that the same institutions continue to publish together with little change to the institutions represented in certain author groups. There is some data to suggest that while the central players in the network, described in part by betweenness scores in Table 3, did not vary greatly across the four issues and continued to published in similar author groups, that some of these institutions formed additional authorship groups with new or existing members of the network. However, these new connections were not brought into the more established authorship groups.

The creation of new authorship groups mentioned above is supported by the fact that the average number of connections per institution increased between the initial and most recent special issue. At the same time, measures of power concentration within the network decreased over the four-year period. This suggests that, aside from traditional key players reaching out to form new groups, new institutions are entering the network with each subsequent year with novel authorship groups. Some of the new connections observed in the network may be due to reasons as various as individuals moving to new institutions, a trainee obtaining a new faculty positions, or novel authors joining the network. One might also hypothesize that the expansion of the network is due to both formal connections generated by work on task forces, work groups, committees, and educational scholarship programs, as well as by informal connections such as colleagues not attached to a specific working group.

To understand the network dynamics better, we informally contacted the authors at institutions with the highest consistent metrics in degree centrality and betweenness to provide insights on a departmental approach to creating successful educational scholarship in an attempt to identify common themes. By contacting these representative institutions, we sought to provide insight on approaches and key strategies in building productive multi-institutional collaborations for educational scholarship.

Based on discussions among the authors regarding the content of these discussions, there were some common threads for collaboration success. The first approach to scholarship was participation in working groups, task forces and longitudinal educational scholarship programs at a national level, such as Medical Education Research Certification at CORD, which appears to be important in developing multi-institutional research.^16^ These successful collaborations started with an author group that was passionate about a specific question and topic. Second, after working together on smaller projects, relationships and research groups formed that then led to working on other papers. These groups changed over time as new people joined and left, and new connections were made. As some research groups matured, collaborators brought in new members leading to new ideas and an organic growth of the network. Finally, sometimes groups have a strong educational researcher or mentor that helps to drive the work and provides opportunities for others to engage.

## LIMITATIONS

Limitations of this study included inability to account for changes made by the movement of people to new institutions. It is unclear how these movements may have affected the yearly rankings based on the data from the above figures. Additionally, this SNA is a snapshot of one journal and its special issue. The *West*JEM Special Issue in Educational Research and Practice is co-sponsored by CORD, which may lead to a bias in how collaborations are created (eg, meeting at the annual CORD assembly). Another significant limitation was the potential publication bias by the supplement in the choice to publish specific manuscripts. Although some of the process may be blinded, the reviewers and editors may have their own biases regarding which types of articles they choose.

Additional research is needed to identify how research networks are formed for publications of other journals. Future research is needed to further our understanding of how network connections and academic collaborations are forged, and the factors – whether individual, institutional, or across a network such as that described here – may lead to more and stronger connections among academic educators. The time covered by this analysis, four issues of one journal in four different years, may not be sufficient to detect changes that require a greater amount of time, eg, changes resulting from key authors changing, changes in leadership, or changes to the practice environment.

## CONCLUSION

By performing a social network analysis of the *West*JEM Special Issue in Educational Research and Practice, we sought to identify patterns of collaboration within the institutional authorship groups and, additionally, to understand which institutions were consistently high performers in terms of connectedness and centrality within the network. This social network analysis provides insight into the early network created by the initial four years of the special issue. Future work is required to determine whether these findings are consistent across other journals (generalizable) and whether or not changes take place in the network that were not identified by this study due to a limited period.

## Figures and Tables

**Table 1:** Definitions of selected social network metrics included in this study assessing connectivity among authors and institutions.

Network level metrics	
Average degrees	The average number of connections for a member of the network. This helps describe how connected an average (typical) institution is across the special issue network.
Network density	The proportion of actual connections to all possible connections across the entire special issue network (range: 0–1). In the context of this study, a denser network (higher value, closer to 1) would mean the authors’ institutions are more directly connected to each other, while a less dense network (closer to 0) would mean fewer direct connections between author institutions making up the special issue network.^10^
Degree centralization	Measures the concentration of power or influence within a network or the variance in the distribution of centrality in a network. This is a normalized value of the importance of single players within the given network. In our case, high degree centralization would suggest that the network is characterized by few centralized institutions whereas a low centralization score would suggest that institutions are more evenly distributed across the special issue network.

Node level metrics	

Degree centrality	The number of connections between one institution and the other institutions within the network. In this study, a network node is represented by a single institution and the degree would count the number of connections to other institutions making up the special issue network.^11^
Betweenness centrality	Measure of how often a node (institution) is connected to other nodes (institutions) that are not then connected to each other. As such, the measure serves as an indicator of which institutions serve as key bridges or connectors within the special issue network.^10^,^11^

**Table 2:** Whole-network metrics for each year of the Special Issue in Educational Research and Practice and all years combined.

Network metrics	2015	2017	2018	2019	All years
Density	0.08	0.1	0.09	0.1	0.08
Average degree	3.48	6.1	5.61	6.17	9.66
Degree centralization	0.31	0.33	0.23	0.24	0.32
Authors from two+ institutions (%)	42.42	69.44	55.56	54.84	55.88

**Table 3:** Degree centrality and betweenness metrics for select institutions in each year and cumulatively.

Degree centrality (rank)								

	2015	2017	2018	2019	All years	# Publications^1^	NIH rank^2^	Program length (years)
Average degree centrality	3.48	6.1	5.61	6.17	9.66			
Michigan	17 (1st)	20 (2nd)	24 (1st)	11 (11th)	73	24	1st	4
Mt. Sinai	8 (2nd-tie)	16 (3rd)	19 (4th)	23 (1st)	66	12	4th	4
Ohio State	3 (24th)	8 (19th-tie)	20 (2nd-tie)	9 (18th-tie)	41	17	15th	3
University of Washington	8 (2nd-tie)	1 (53rd-tie)	10 (10th-tie)	8 (25th-tie)	32	6	Not ranked	4
Yale	8 (2nd-tie)	5 (30th-tie)	n/a	16 (6th)	29	6	3rd	4

Betweenness (rank)								

Michigan	0.30 (1st)	0.12 (3rd)	0.14 (1st)	0.02 (12th)	0.12	24	1st	4
Mt. Sinai	0.03 (6th)	0.16 (2nd)	0.06 (4th)	0.06 (2nd)	0.12	12	4th	4
Ohio State	0.03 (7th)	0.02 (14th)	0.06 (5th)	0.06 (3rd)	0.08	17	15th	3
University of Washington	0.15 (2nd)	0 (27th-tie)	0 (13th-tie)	0 (16th-tie)	0.06	6	Not ranked	4
Yale	0.03 (9th)	0 (27th-tie)	n/a	0.09 (1st)	0.05	6	3rd	4

1 This is the number of publications in the dataset for Social network analysis.

2 NIH (National Institutes of Health) research rankings provides a benchmark for other research in the department (http://www.brimr.org/NIH_Awards/2018/NIH_Awards_2018.htm).
